# The Value of Platelet-to-Lymphocyte Ratio as a Prognostic Marker in Cholangiocarcinoma: A Systematic Review and Meta-Analysis

**DOI:** 10.3390/cancers14020438

**Published:** 2022-01-16

**Authors:** Dong Liu, Zoltan Czigany, Lara R. Heij, Stefan A. W. Bouwense, Ronald van Dam, Sven A. Lang, Tom F. Ulmer, Ulf P. Neumann, Jan Bednarsch

**Affiliations:** 1 Department of Surgery and Transplantation, University Hospital RWTH Aachen, 52074 Aachen, Germany; dliu@ukaachen.de (D.L.); zczigany@ukaachen.de (Z.C.); lheij@ukaachen.de (L.R.H.); svlang@ukaachen.de (S.A.L.); fulmer@ukaachen.de (T.F.U.); 2 Institute of Pathology, University Hospital RWTH Aachen, 52074 Aachen, Germany; 3 Department of Surgery, Maastricht University Medical Center (MUMC), 6229 HX Maastricht, The Netherlands; stefan.bouwense@mumc.nl (S.A.W.B.); r.van.dam@mumc.nl (R.v.D.)

**Keywords:** cholangiocarcinoma (CCA), platelet-to-lymphocyte ratio (PLR), oncological prognosis, systematic review, meta-analysis

## Abstract

**Simple Summary:**

Platelet-to-lymphocyte ratio has shown prognostic value in several malignancies; however, its role in cholangiocarcinoma remains to be determined. Therefore, we conducted a systematic review and meta-analysis of the currently available literature. Overall, our analysis revealed that a high platelet-to-lymphocyte ratio before treatment is associated with an impaired long-term oncological outcome. Further, our results indicate that this assumption was not influenced by the used treatment modality (surgical vs. non-surgical), PLR cut-off values, study population age, or sample size of the included studies. Thus, an elevated pretreatment platelet-to-lymphocyte ratio has valid prognostic value for cholangiocarcinoma patients.

**Abstract:**

The platelet-to-lymphocyte ratio (PLR), an inflammatory parameter, has shown prognostic value in several malignancies. The aim of this meta-analysis was to determine the impact of pretreatment PLR on the oncological outcome in patients with cholangiocarcinoma (CCA). A systematic literature search has been carried out in the PubMed and Google Scholar databases for pertinent papers published between January 2000 and August 2021. Within a random-effects model, the pooled hazard ratio (HR) and 95% confidence interval (CI) were calculated to investigate the relationships among the PLR, overall survival (OS), and disease-free survival (DFS). Subgroup analysis, sensitivity analysis, and publication bias were also conducted to further evaluate the relationship. A total of 20 articles comprising 5429 patients were included in this meta-analysis. Overall, the pooled outcomes revealed that a high PLR before treatment is associated with impaired OS (HR = 1.14; 95% CI = 1.06–1.24; *p* < 0.01) and DFS (HR = 1.57; 95% CI = 1.19–2.07; *p* < 0.01). Subgroup analysis revealed that this association is not influenced by the treatment modality (surgical vs. non-surgical), PLR cut-off values, or sample size of the included studies. An elevated pretreatment PLR is prognostic for the OS and DFS of CCA patients. More high-quality studies are required to investigate the pathophysiological basis of the observation and the prognostic value of the PLR in clinical management as well as for patient selection.

## 1. Introduction

Cholangiocarcinoma (CCA) is the second most common primary liver tumor, accounting for 5 to 30% of all primary liver malignancies. It originates from mutated epithelial cells of the hepatic bile ducts [1,2]. With respect to the anatomical location, CCA can be divided into intrahepatic CCA (iCCA) and extrahepatic CCA (eCCA), which are also related to distinct pathophysiology and clinical outcomes [3,4].

The inflammatory response of the host in the tumor microenvironment is known to play a crucial role in cancer growth and progression and is further linked to systemic inflammation [5]. In this context, counts of neutrophils, lymphocytes, and platelets, as well as hypoalbuminemia and high C-reactive protein (CRP) levels have all been used to calculate clinical scores or ratios, such as the Glasgow Prognostic Score (GPS), the platelet-to-lymphocyte ratio (PLR), and the neutrophil-to-lymphocyte ratio (NLR), which have shown associations with oncological and surgical outcomes in various solid tumors [6,7,8,9]. However, there are conflicting results regarding these preoperative systemic inflammatory parameters in CCA [10,11,12].

Thrombocytosis is prevalent in patients with solid tumors, indicating an interaction between cancer and platelets [13]. Platelets have been shown to interact directly with tumor cells, releasing substances that aid tumor development, invasion, and angiogenesis and have the ability to protect tumor cells from destruction by natural killer cells [14,15]. In numerous solid tumors, for example, breast, lung, colon, gastric, and ovarian cancer, a relationship between thrombocytosis and an impaired oncological outcome has been demonstrated [16]. As low lymphocyte counts may also be associated with shorter oncological survival, the ratio of platelet to lymphocyte (PLR) has been proposed as a prognostic biomarker [17,18]. The aim of this systemic review and meta-analysis was, therefore, to elucidate the role of PLR in oncological outcomes in CCA.

## 2. Materials and Methods

### 2.1. Literature Search

This systematic review was registered in the International Prospective Register of Systematic Reviews (PROSPERO) with the ID CRD42021271435 and was conducted in accordance with the PRISMA (Preferred Reporting Items for Systematic Reviews and Meta-analyses) criteria. PubMed and Google Scholar were systematically searched. The following full-text terms were searched: “Lymphocytes” OR “Platelet-to-lymphocyte ratio” AND “Cholangiocarcinoma (CCA)” OR “Biliary tract cancers (BTC)”. The Boolean operator “OR” was used to combine all expressions of cases including abbreviation, while “AND” was used to include lymphocytes and PLR in conjunction with CCA in the search. The search period of the electronic database was from January 2000 to October 2021. During the literature search, no proximity operators were used. Two authors (LD and JB) conducted two independent literature searches in this systematic review, both using the same strategy. No additional papers were chosen after the reference list and citation search were completed. There was no search for unpublished literature.

### 2.2. Inclusion and Exclusion Criteria

Studies concerning the prognostic role of the PLR in CCA were the first choice for inclusion. Further criteria for selection included data on overall survival (OS) or disease-free survival (DFS) for evaluation and pre-treatment determination of the PLR. Exclusion criteria were (1) no access to the full text for quality assessment and data extraction; (2) review, case report, comment, or editorial; (3) non-English studies.

### 2.3. Statistical Analysis

The hazard ratio (HR) and its 95% confidence interval (CI) were used to assess the association between the PLR and the prognosis of CCA. If the relevant data was not directly reported, it was extracted using survival data from Kaplan–Meier curves by Engauge Digitizer version 12.1, as described previously [19]. RevMan version 5.4 (Cochrane Collaboration, London, UK) was used to merge the results of the studies. Statistical heterogeneity between trials was assessed by a Chi-squared test and suggested to be significant when I^2^ > 50% and/or *p* < 0.05. A fixed-effects model was used when no heterogeneity was detected among studies, while a random-effects model and subgroup analysis were preferred when variance existed. Subgroup analysis was conducted to explore and explain the heterogeneity among the results of different studies. To determine the stability of the overall treatment effects, sensitivity analyses were performed. Therefore, we excluded one study at a time to ensure that no single study would be solely responsible for the significance of any result. Funnel plots were performed to evaluate publication bias [20].

### 2.4. Quality Assessment of Studies

The quality of the selected studies was systematically evaluated by 2 reviewers (DL and JB) using the Newcastle–Ottawa scale [21]. The Newcastle–Ottawa scale is composed of three parameters of quality—selection, outcome assessment, and comparability. Each study was subsequently scored from 0 to 9 points, with higher values indicating better quality.

## 3. Results

### 3.1. Literature Search

**Figure 1:**
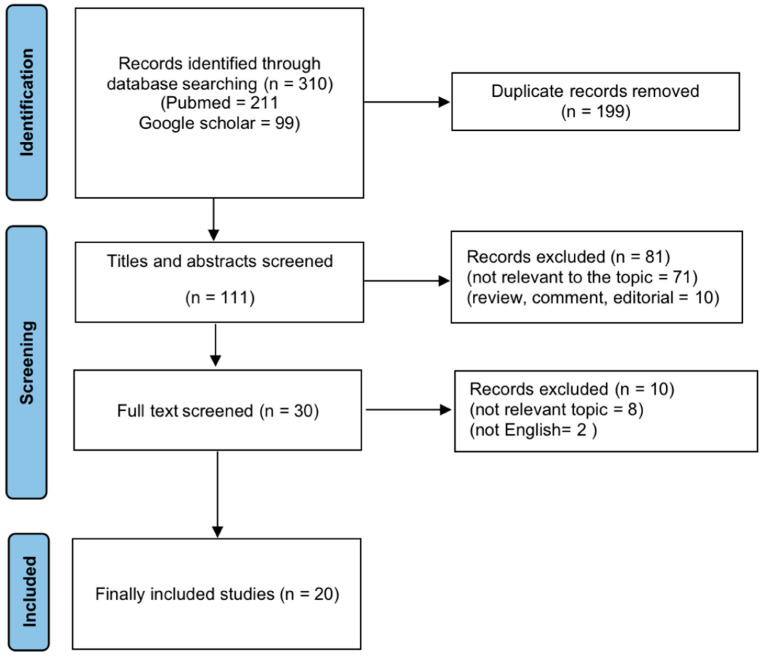
Flowchart of study selection for this study.

The process of selecting articles is depicted in the PRISMA diagram below (Figure 1). A total of 310 articles were found initially after searching the two databases. Then, 199 duplicate records were discovered and eliminated. The remaining 111 studies were further vetted for eligibility after the titles and abstracts were reviewed. Ultimately, 20 studies were included in the analysis [22,23,24,25,26,27,28,29,30,31,32,33,34,35,36,37,38,39,40,41].

### 3.2. Study Characteristics and Quality Assessment

An overview of the included publications is provided in Table 1. All studies were retrospective cohort studies published over the last 6 years. A total of 5429 patients were eligible for analysis, of which 4453 individuals underwent liver resection, while 976 were treated non-surgically. Each study reported survival and the PLR using a variety of approaches. A subset of 12 studies investigated iCCA, 6 studies focused on eCCA, and 2 studies comprised both tumor entities. All studies reported a correlation between OS and the PLR, and 8 studies further reported a correlation between DFS and the PLR. The Newcastle–Ottawa scale ranged from 6 to 9, indicating an overall good quality of the methodology of the included studies (Table 2).

### 3.3. Correlation between the PLR and OS of CCA Patients

**Figure 2:**
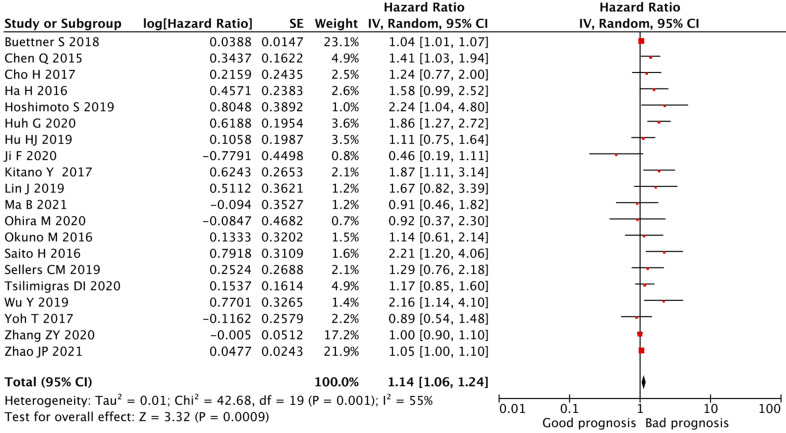
**Forest plot of the correlation between PLR and OS in CCA patients.** A random-effects model was used to estimate the relationship between PLR and OS. OS, overall survival; PLR, platelet-to-lymphocyte ratio.

Eight studies identified the PLR as an independent predictor for impaired OS in patients with CCA [22,28,29,33,34,36,38,41], while the PLR was not prognostic for OS in twelve studies [23,24,25,26,27,30,31,32,35,37,39,40]. The combined analysis of all twenty publications showed that the PLR values higher than the defined cut-off values predicted a worse OS (HR = 1.14, 95% CI = 1.06–1.24, *p* < 0.01) with high heterogeneity (I^2^ = 55%, *p* < 0.01, Figure 2).

### 3.4. Correlation between the PLR and DFS of CCA Patients

**Figure 3:**
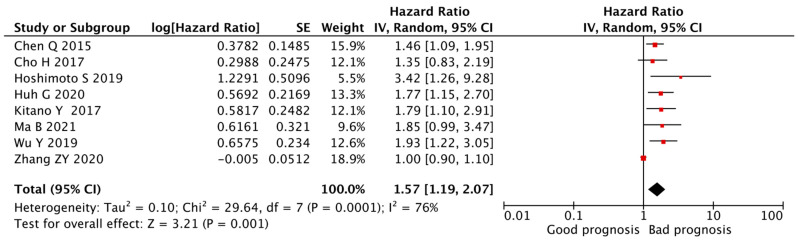
**Forest plot of the correlation between PLR and DFS in CCA patients.** A random-effects model was used to estimate the relationship between PLR and DFS. DFS, disease-free survival; PLR, platelet-to-lymphocyte ratio.

Five cohort studies showed that the PLR was an independent indicator of poor DFS in patients with CCA [28,29,31,32,36], whereas three publications detected no significant relationship between the PLR and DFS [22,23,36]. The pooled analysis of all eight studies revealed that a higher PLR was associated with worse DFS (HR = 1.57, 95% CI = 1.19–2.07, *p* < 0.01) with high heterogeneity (I^2^ = 76%, *p* < 0.01, Figure 3).

### 3.5. Subgroup Analyses of Correlation between the PLR and OS of CCA Patients

As stated above, significant heterogeneity was observed in the HR of the OS for the PLR (I2 = 55%, *p* < 0.01, Figure 2) and the DFS for the PLR (I2 = 76%, *p* < 0.01, Figure 3). Considering the limited number of studies of DFS, we exclusively explored potential causes of the heterogeneity of the OS by subgroup analyses, focusing on cancer type, specific treatment, PLR cut-off values, sample size, and age.

First, we analyzed the significance of a high PLR with respect to OS for patients according to different cancer types, including CCA (combined analysis of iCCA and eCCA), iCCA, and eCCA. While statistical heterogeneity was observed in the eCCA subgroup (I2 = 60%, *p* = 0.03), with no significant correlation to OS, heterogeneity was not found in the CCA subgroup (I2 = 0%, *p* = 0.44) or the iCCA subgroup (I2 = 35%, *p* = 0.12, Table 3, Figure S1A).

Between seventeen surgical and three non-surgical studies, the prognostic role of the PLR in OS was unfavorable in both subgroups (surgery group: HR = 1.09, 95% CI = 1.02–1.17, *p* = 0.02; non-surgical group: HR = 1.58, 95%, CI = 1.23–2.04, *p* = 0.0003). Of note, heterogeneity was not found to be significant in this subgroup analysis (surgery group: I^2^ = 48%, *p* = 0.01; non-surgical group: I^2^ = 0%, *p* = 0.43, Table 3, Figure S1B).

Because the PLR cut-off values were certainly different among the studies, ranging from 90 to 270, we performed further subgroup analysis based on the PLR cut-off value. In 11 studies with a PLR cut-off greater than 150, and 9 studies with a PLR of less than 150, the pooled analysis of the PLR with respect to OS was significant in both subgroups (PLR ≥ 150 group: HR = 1.17, 95% CI = 1.02–1.33, *p* = 0.02; PLR < 150 group: HR = 1.25, 95% CI = 1.03–1.51, *p* = 0.02). Statistical heterogeneity was found in the subgroup with a PLR ≥150 (I^2^ = 60%, *p* < 0.01) and in the subgroup with a PLR < 150 (I^2^ = 53%, *p* = 0.03, Table 3, Figure S1C).

We further divided the 20 studies into two distinct subgroups according to the sample size (≥200 and <200 cases). Both groups showed significance regarding the prognostic value of the PLR (sample size ≥200: HR = 1.07, 95% CI = 1.01–1.13, *p* = 0.02; sample size <200: HR=1.38, 5% CI = 1.07–1.77, *p* = 0.01). However, statistical heterogeneity was not found in the subgroup with a sample size ≥ 200 (I2 = 32%, *p* = 0.15) but was found in the other corresponding subgroup with a sample size <200 (I2 = 69%, *p* = 0.01, Table 3, Figure S1D).

With respect to age, two groups (median or mean age ≥60 and <60) were analyzed and showed low heterogeneity (I2 = 36%, *p* = 0.13 and I2 = 40%, *p* = 0.08). The HRs for the OS for age ≥60 years and for age <60 years were 1.48 (95% CI = 1.18–1.85, *p* < 0.01) and 1.06 (95% CI = 1.00–1.12, *p* = 0.05), respectively (Table 3, Figure S1E).

### 3.6. Sensitivity Analyses of Correlation between the PLR and Prognosis of CCA Patients

**Figure 4:**
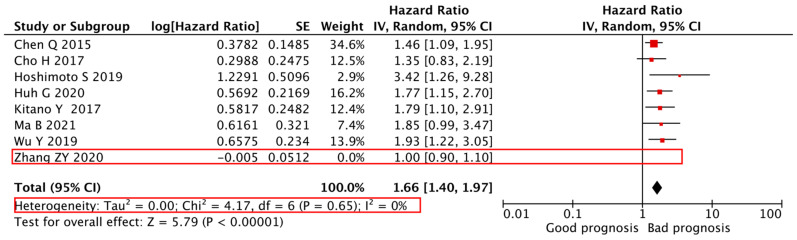
**Sensitivity analyses of the association between thePLR and DFS in CCA patients.** Sensitivity analyses of the association between the PLR and DFS of 8 studies. A random-effects model was used. DFS, disease-free survival; PLR, platelet-to-lymphocyte ratio.

**Figure 5:**
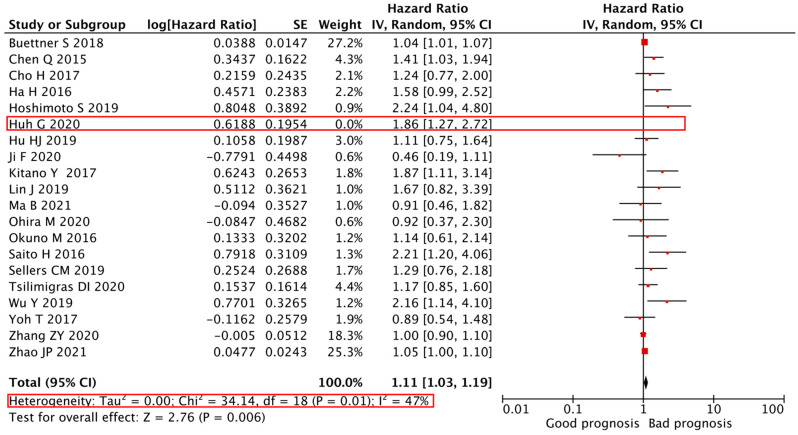
**Sensitivity analyses of the association between thePLR andOS in CCA patients.** Sensitivity analyses of the association between the PLR and OS of 20 studies. A random-effects model was used. OS, overall survival; PLR, platelet-to-lymphocyte ratio.

We adopted a random-effects model in the sensitivity analyses, deleting each study in each turn, to further determine the robustness of the prognostic role of the PLR in the DFS and OS of CCA. As shown in Figure 4 and Figure 5, the results of heterogeneity were changed when the studies of Zhang Z.Y. et al. [24] and Huh G. et al. [28] were deleted from the data set. The I^2^ decreased from 76 to 0% and 55 to 47%, respectively, but still displayed an unfavorable prognostic effect for DFS (HR = 1.66, 95% CI = 1.40–1.97, *p* < 0.01) and OS (HR = 1.11, 95% CI = 1.03–1.19, *p* < 0.01). These results indicate a significant contribution of these studies to the high heterogeneity regarding the outcome measures and further support the robustness of the PLR as a prognostic factor of DFS and OS.

### 3.7. Publication Bias

The results from the funnel plot analysis (Figure S2) demonstrated that asymmetry was not present and, therefore, no publication bias affecting the HRs could be displayed.

## 4. Discussion

According to meta-analyses on several malignancies, including head and neck [42], lung [43], breast [44], renal [45], prostate [46], esophageal [47], pancreatic [48], colorectal [49], and hepatocellular cancers [50], high PLR values are associated with poor oncological survival. In contrast, the PLR has also been shown to be an unreliable prognostic predictor in patients in some other scenarios, for example, gastric cancer [51]. In the present meta-analysis of 20 studies comprising 5429 patients with CCA, we were able to demonstrate that the PLR is of prognostic value in CCA patients. The pooled outcomes revealed that a high pretreatment PLR is associated with impaired OS (HR = 1.14, 95% CI = 1.06–1.24, *p* < 0.01) and reduced DFS (HR = 1.57, 95% CI = 1.19–2.07, *p* < 0.01).

Of note, our subgroup analysis further demonstrated that the unfavorable effect of the PLR is independent from different treatment types, including palliative or curative therapy, the sample size, and the PLR cut-off value, while we observed no statistical significance for patients younger than 60 years. The independence of the prognostic value from the used treatment is particularly interesting, as the genuine oncological outcome of palliative compared to curative treatment is hardly comparable in CCA. While the median OS in the palliative setting is usually less than 12 months with systemic therapy, 5-year survival rates higher than 50% are reported in distinct subgroups of CCA patients after curative-intent surgical therapy [52,53,54]. This observation indicates that the PLR could be closely associated with the individual tumor biology and, therefore, could predict the outcome irrespective of the standard of care treatment at each oncological stage. Interestingly, Zheng et al. conducted a similar meta-analysis for hepatocellular carcinoma and also identified a high PLR as an independent risk factor for OS and DFS in HCC patients, both within curative and palliative settings [50].

Further, our subgroup analysis failed to detect a statistically significant association between OS and the PLR in eCCA patients with marginally non-significant values (HR = 1.37, 95% CI = 0.93–2.03, *p* = 0.11). However, it must be noted that the number of included eCCA studies was limited (n = 6) and comprised perihilar as well as distal cholangiocarcinoma, translating to significant heterogeneity (I^2^ = 60%, *p* = 0.03. Figure S1A, Table 3) between the included studies. In addition, eCCA is characterized by a high degree of infectious complications due to recurrent cholangitis, which might be the cause of a dismal long-term outcome [55,56]. Considering the primary association of the PLR with oncological survival, it can be assumed that the predictive value of the PLR might be mitigated by the impaired outcome due to septic events.

Chronic inflammation is thought to play a major role in up to 15% of cancer cases around the world [57]. It is generally known that the systemic inflammatory response plays a key role in carcinogenesis and patient survival. Systemic inflammation is primarily reflected by changes in blood parameters and can be determined by the number of several cell components (neutrophils, lymphocytes, monocytes, and platelets) using standard clinical thresholds [58]. Tumor cells have been demonstrated to excrete platelet-stimulating factors, which promote primary tumor growth, invasion, and metastasis through a variety of pathways [59]. As a result, the platelet count in the peripheral blood is suggested to be an indirect predictor of tumor activity [60,61]. Moreover, the presence of antitumor lymphocytes in the peripheral blood, particularly CD8+ T cells, would indicate tumor suppression activity [62]. The ratio of platelets and lymphocytes could, therefore, be an indicator of antitumor activity, prognosis, and/or response to treatment.

However, the detailed mechanism behind the prognostic significance of the PLR in cancer is still unknown. Cancer and inflammatory cells interact reciprocally in experimental studies in terms of extracellular matrix remodeling, angiogenesis, and metastatic preparation [63]. An elevated PLR indicates the activation of transcription factors of an inflammatory response, for example, the signal transducer and activator of transcription 3 (STAT3), hypoxia-inducible factor 1a (HIF1a), and nuclear factor-kB (NF-kB) [64,65]. These transcription factors result in the secretion of pro-inflammatory cytokines that also promote tumor growth, such as TNF-a, IL-1β, and IL-6 [66,67]. In addition, cancer-related inflammation plays a role in epithelial–mesenchymal transition (EMT), angiogenesis, cell proliferation and survival, tumor–cell migration, invasion, and metastasis, as well as treatment response [63]. NF-κB was found to be overexpressed in CCA tissues and the inhibition of NF-κB action significantly enhanced cell apoptosis and reduced cell growth, which suggests NF-κB as a potential molecular target for CCA therapy [68]. Yang et al. observed that STAT3 overexpression promotes metastasis in iCCA and correlates negatively with surgical outcomes [69]. In addition, Yu et al. found that HIF1a could activate the lncRNA H19-mediated miR-612/Bcl-2 pathway to promote cholangiocarcinoma [70]. An elevated PLR might, therefore, also be a surrogate for the activity of transcription factors associated with cancer progression in CCA. This is also supported by an increasing number of studies focusing on the relationship between the PLR and tumor characteristics. For example, in cervical cancer and colorectal cancer, a higher PLR was associated with a higher rate of lymph node metastasis [71,72].

It is well known that escaping from immune surveillance is a hallmark feature of tumorigenesis and cancer progression. Recently, immunotherapy, for example, immune checkpoint inhibitors (ICIs), chimeric antigen receptor (CAR) T-cells, and tumor vaccines emerged as novel treatment modalities for malignancies [73]. However, the response rates to immunotherapy are quite different in CCA compared to other solid tumors due to the spatial heterogeneity of biliary tract cancer itself [74]. In fact, there is a lack of reliable predictive biomarkers, which is a main obstacle in the use of immunotherapies in CCA [75]. Thus, the identification of such biomarkers identifying subgroups would facilitate clinical management in CCA significantly.

A recent meta-analysis of 1845 patients with non-small cell lung cancer (NSCLC) comprising 21 studies that included treatment with three ICIs found that a high PLR was associated with poor OS and PFS [76]. Another meta-analysis from Xu et al. evaluated 12 trials including 1430 cancer patients and observed the detrimental impact a high PLR had on the efficacy of ICIs (HR for OS = 2.0) [77]. Diem et al. also reported that an elevated PLR was associated with shorter OS and PFS and lower response rates in NSCLC patients treated with nivolumab [78]. Hence, the PLR might serve as an easily accessible prognostic marker for the response to immunotherapy. Nevertheless, Zer et al. detected no significant link between the baseline PLR and immunotherapy efficacy in CCA. Therefore, more studies are required to evaluate the PLR as a predictive biomarker for monitoring therapy success [79].

As with all meta-analyses with limited available literature, our study has certain limitations. All included studies were retrospective analyses in nature, leaving a potential selection bias in the published data. In addition, a variety of methodologies was used and, most importantly, different PLR cut-off levels. These different cut-offs impede the routine application of the parameter in clinical management and warrant further research. As some studies did not report HRs and CIs in detail, these variables had to be extrapolated from the survival curves in four studies [24,25,26,27,28,29,30,31,37,39]. Further, the available data were unfortunately not sufficient to investigate the association between the PLR and tumor clinicopathological characteristics.

## 5. Conclusions

In light of the above, we were able to demonstrate that an elevated pretreatment PLR is predictive for the prognosis of CCA patients. Large-scale prospective cohort studies are warranted to confirm the independent prognostic effect of the PLR on CCA.

## Figures and Tables

**Table 1:** **Characteristics of the 20 eligible studies evaluating PLR in CCA.** CCA, cholangiocarcinoma; DFS, disease-free survival; ECCA, extrahepatic cholangiocarcinoma; ICCA, intrahepatic cholangiocarcinoma; NR, not reported; OS, overall survival; PLR, platelet-to-lymphocyte ratio; Ref, reference.

Ref.	Author	Year Published	Country	Tumor Type	Sample Size	Stage	Age (Median)	Male (%)	Treatment	Follow-Up (Months, Median)	Endpoint	Cut-Off Value
[22]	Zhao JP	2021	China	ICCA	468	NR	58	60.30%	Surgery	NR	OS	PLR ≥ 143.5
[23]	Ma B	2021	China	ICCA	174	I–IV	58	55.90%	Surgery	25.1	OS/DFS	PLR ≥ 90
[24]	Zhang ZY	2020	China	ICCA	128	I–III	56	55.00%	Surgery	NR	OS/DFS	PLR ≥ 156.8
[25]	Tsilimigras DI	2020	USA	ICCA	688	I–III	57	60.50%	Surgery	22.3	OS	PLR ≥ 190
[26]	Ohira M	2020	Japan	ICCA	52	I–IV	58	78.84%	Surgery	NR	OS	PLR ≥ 98
[27]	Ji F	2020	China	ECCA	59	I–IV	57	55.93%	Surgery	NR	OS	PLR ≥ 268.9
[28]	Huh G	2020	Korea	ICCA	137	III–IV	64	60.60%	Non-surgery	9.9	OS/DFS	PLR ≥ 148
[29]	Wu Y	2019	China	CCA	119	NR	60	42.90%	Surgery	11	OS/DFS	PLR ≥ 157.3
[30]	Sellers CM	2019	USA	ICCA	131	I–IV	65	51.90%	Surgery	13	OS	PLR ≥ 156.4
[31]	Lin J	2019	China	ICCA	218	I–IV	60	56.90%	Surgery	NR	OS	PLR ≥ 130.6
[32]	Hu HJ	2019	China	ECCA	134	I-IV	60	63.01%	Surgery	NR	OS	PLR ≥ 150
[33]	Hoshimoto S	2019	Japan	ECCA	53	I–IV	70	58.00%	Surgery	18	OS/DFS	PLR ≥ 187.8
[34]	Buettner S	2018	Netherlands	ICCA	991	I–IV	59	54.10%	Surgery	29	OS	PLR ≥ 190
[35]	Yoh T	2017	Japan	ICCA	141	I–IV	65	63.00%	Surgery	NR	OS	PLR ≥ 120
[36]	Kitano Y	2017	Japan	ECCA	120	I–IV	58	68.33%	Surgery	NR	OS/DFS	PLR ≥ 185
[37]	Cho H	2017	Korea	ICCA	305	III–IV	59	61.50%	Non-surgery	25	OS/DFS	PLR ≥ 128.3
[38]	Saito H	2016	Japan	ECCA	121	I–IV	70	72.72%	Surgery	NR	OS	PLR ≥ 150
[39]	Okuno M	2016	Japan	ECCA	534	I-IV	66	62.92%	Surgery	78	OS	PLR ≥ 150
[40]	Ha H	2016	Korea	CCA	534	III–IV	60	65.20%	Non-surgery	95.3	OS	PLR ≥ 89.6
[41]	Chen Q	2015	China	ICCA	322	I–IV	58	60.25%	Surgery	NR	OS/DFS	PLR ≥ 123

**Table 2:** **Quality of included cohort studies evaluated by modified Newcastle–Ottawa scale.** The quality of the included studies was assessed under six items of Hayden et al. [21] All included translational studies reporting oncological outcomes were evaluated in accordance with the Newcastle–Ottawa scale. The maximum score of the scale is nine points, with studies being categorized as low (0–3 points), moderate (4–6 points), or high quality (7–9 points).

Ref.	Author	Selection	Comparability	Outcomes	Quality Score
[22]	Zhao JP	★★★★	★★	★★	9
[23]	Ma B	★★★	★★	★★	8
[24]	Zhang ZY	★★★★	★★	★★	9
[25]	Tsilimigras DI	★★★★	★★	★★	9
[26]	Ohira M	★★★★	★★	★★	9
[27]	Ji F	★★★★	★★	★★	9
[28]	Huh G	★★★★	★★	★★	9
[29]	Wu Y	★★★★	★★	★★	9
[30]	Sellers CM	★★★★	★★	★★	9
[31]	Lin J	★★★★	★★	★★	9
[32]	Hu HJ	★★★	★★	★★	8
[33]	Hoshimoto S	★★★	★★	★★	8
[34]	Buettner S	★★★★	★★	★	8
[35]	Yoh T	★★★	★★	★	6
[36]	Kitano Y	★★★★	★★	★★	9
[37]	Cho H	★★★★	★★	★	8
[38]	Saito H	★★★★	★★	★★	9
[39]	Okuno M	★★★★	★★	★★	9
[40]	Ha H	★★★★	★★	★★	9
[41]	Chen Q	★★★★	★★	★★	9

**Table 3:** **Summary of the subgroup analyses of the correlation between the PLR and OS in CCA patients.** * Includes both ICCA and ECCA. ** Mean/median age of the study cohort. ECCA, extrahepatic cholangiocarcinoma; ICCA, intrahepatic cholangiocarcinoma; NLR, neutrophil-to-lymphocyte ratio; PLR, platelet-to-lymphocyte ratio.

Subgroup	Number of Studies	HR (95% CI)	*p* Value	Heterogeneity	
				I^2^	*p*
**Cancer type**					
CCA *	2	1.76 (1.21–2.57)	<0.01	0%	0.44
ICCA	12	1.06 (1.00–1.12)	0.03	35%	0.12
ECCA	6	1.37 (0.93–2.03)	0.11	60%	0.03
**Treatment**					
Surgery	17	1.09 (1.02–1.17)	0.02	48%	0.01
Non-surgery	3	1.58 (1.23–2.04)	<0.01	0%	0.43
**Cut-off value**					
PLR ≥ 150	11	1.17 (1.02–1.33)	0.02	60%	<0.01
PLR < 150	9	1.25 (1.03–1.51)	0.02	53%	0.03
**Sample size**					
≥200	10	1.07 (1.01–1.13)	0.02	32%	0.15
<200	10	1.38 (1.07–1.77)	0.01	69%	0.01
Age **					
≥60	9	1.48 (1.18–1.85)	<0.01	36%	0.13
<60	11	1.06 (1.00–1.12)	0.05	40%	0.08

## Data Availability

The data presented in this study are available upon request from the corresponding author.

## Supplementary Materials

The following are available online at https://www.mdpi.com/article/10.3390/cancers14020438/s1, Figure S1: Stratified forest plots of the association between the PLR and OS in CCA patients. Figure S2: Funnel plot of the meta-analysis of PLR in OS (A) and DFS (B).
