# Supplementary material for: The Value of Platelet-to-Lymphocyte Ratio as a Prognostic Marker in Cholangiocarcinoma: A Systematic Review and Meta-Analysis
Source: Cancers (Basel). 2022 Jan 16;14(2):438. doi: 10.3390/cancers14020438 (PMC8773915; doi:10.3390/cancers14020438)
Supplement: Supplementary file 1 [file cancers-14-00438-s001.zip › cancers-1533230-supplementary.pdf]

A

| Study or Subgroup | log[Hazard Ratio] | SE | Weight | Hazard Ratio IV, Random, 95% CI |
|---|---|---|---|---|
| **1.3.1 CCA** | | | | |
| Ha H 2016 | 0.4571 | 0.2383 | 65.2% | 1.58 [0.99, 2.52] |
| Wu Y 2019 | 0.7701 | 0.3265 | 34.8% | 2.16 [1.14, 4.10] |
| **Subtotal (95% CI)** | | | **100.0%** | **1.76 [1.21, 2.57]** |
| Heterogeneity: $Tau^2 = 0.00$; $Chi^2 = 0.60$, df = 1 (P = 0.44); $I^2 = 0\%$ | | | | |
| Test for overall effect: Z = 2.94 (P = 0.003) | | | | |
| **1.3.2 ICCA** | | | | |
| Buettner S 2018 | 0.0388 | 0.0147 | 39.3% | 1.04 [1.01, 1.07] |
| Chen Q 2015 | 0.3437 | 0.1622 | 2.6% | 1.41 [1.03, 1.94] |
| Cho H 2017 | 0.2159 | 0.2435 | 1.2% | 1.24 [0.77, 2.00] |
| Huh G 2020 | 0.6188 | 0.1954 | 1.8% | 1.86 [1.27, 2.72] |
| Ma B 2021 | −0.094 | 0.3527 | 0.6% | 0.91 [0.46, 1.82] |
| Ohira M 2020 | −0.0847 | 0.4682 | 0.3% | 0.92 [0.37, 2.30] |
| Sellers CM 2019 | 0.2524 | 0.2688 | 1.0% | 1.29 [0.76, 2.18] |
| Tsilimigras DI 2020 | 0.1537 | 0.1614 | 2.6% | 1.17 [0.85, 1.60] |
| Yoh T 2017 | −0.1162 | 0.2579 | 1.1% | 0.89 [0.54, 1.48] |
| Zhang ZY 2020 | −0.005 | 0.0512 | 17.0% | 1.00 [0.90, 1.10] |
| Zhao JP 2021 | 0.0477 | 0.0243 | 32.6% | 1.05 [1.00, 1.10] |
| **Subtotal (95% CI)** | | | **100.0%** | **1.06 [1.00, 1.12]** |
| Heterogeneity: $Tau^2 = 0.00$; $Chi^2 = 15.31$, df = 10 (P = 0.12); $I^2 = 35\%$ | | | | |
| Test for overall effect: Z = 2.13 (P = 0.03) | | | | |
| **1.3.3 ECCA** | | | | |
| Hoshimoto S 2019 | 0.8048 | 0.3892 | 13.7% | 2.24 [1.04, 4.80] |
| Hu HJ 2019 | 0.1058 | 0.1987 | 22.3% | 1.11 [0.75, 1.64] |
| Ji F 2020 | −0.7791 | 0.4498 | 11.6% | 0.46 [0.19, 1.11] |
| Kitano Y 2017 | 0.6243 | 0.2653 | 19.0% | 1.87 [1.11, 3.14] |
| Okuno M 2016 | 0.1333 | 0.3202 | 16.5% | 1.14 [0.61, 2.14] |
| Saito H 2016 | 0.7918 | 0.3109 | 16.9% | 2.21 [1.20, 4.06] |
| **Subtotal (95% CI)** | | | **100.0%** | **1.37 [0.93, 2.03]** |
| Heterogeneity: $Tau^2 = 0.14$; $Chi^2 = 12.64$, df = 5 (P = 0.03); $I^2 = 60\%$ | | | | |
| Test for overall effect: Z = 1.60 (P = 0.11) | | | | |

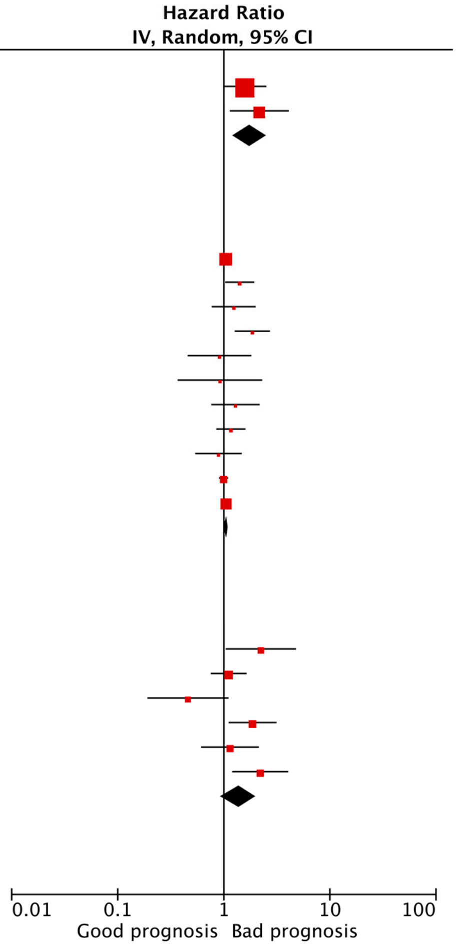

B

| Study or Subgroup | log[Hazard Ratio] | SE | Weight | Hazard Ratio IV, Random, 95% CI |
|---|---|---|---|---|
| **1.2.1 Non-surgical** | | | | |
| Cho H 2017 | 0.2159 | 0.2435 | 27.8% | 1.24 [0.77, 2.00] |
| Ha H 2016 | 0.4571 | 0.2383 | 29.0% | 1.58 [0.99, 2.52] |
| Huh G 2020 | 0.6188 | 0.1954 | 43.2% | 1.86 [1.27, 2.72] |
| **Subtotal (95% CI)** | | | **100.0%** | **1.58 [1.23, 2.04]** |
| Heterogeneity: Tau$^2$ = 0.00; Chi$^2$ = 1.67, df = 2 (P = 0.43); I$^2$ = 0% | | | | |
| Test for overall effect: Z = 3.58 (P = 0.0003) | | | | |
| **1.2.2 Surgery** | | | | |
| Buettner S 2018 | 0.0388 | 0.0147 | 29.1% | 1.04 [1.01, 1.07] |
| Chen Q 2015 | 0.3437 | 0.1622 | 4.3% | 1.41 [1.03, 1.94] |
| Hoshimoto S 2019 | 0.8048 | 0.3892 | 0.9% | 2.24 [1.04, 4.80] |
| Hu HJ 2019 | 0.1058 | 0.1987 | 3.0% | 1.11 [0.75, 1.64] |
| Ji F 2020 | −0.7791 | 0.4498 | 0.6% | 0.46 [0.19, 1.11] |
| Kitano Y 2017 | 0.6243 | 0.2653 | 1.8% | 1.87 [1.11, 3.14] |
| Lin J 2019 | 0.5112 | 0.3621 | 1.0% | 1.67 [0.82, 3.39] |
| Ma B 2021 | −0.094 | 0.3527 | 1.0% | 0.91 [0.46, 1.82] |
| Ohira M 2020 | −0.0847 | 0.4682 | 0.6% | 0.92 [0.37, 2.30] |
| Okuno M 2016 | 0.1333 | 0.3202 | 1.2% | 1.14 [0.61, 2.14] |
| Saito H 2016 | 0.7918 | 0.3109 | 1.3% | 2.21 [1.20, 4.06] |
| Sellers CM 2019 | 0.2524 | 0.2688 | 1.7% | 1.29 [0.76, 2.18] |
| Tsilimigras DI 2020 | 0.1537 | 0.1614 | 4.4% | 1.17 [0.85, 1.60] |
| Wu Y 2019 | 0.7701 | 0.3265 | 1.2% | 2.16 [1.14, 4.10] |
| Yoh T 2017 | −0.1162 | 0.2579 | 1.9% | 0.89 [0.54, 1.48] |
| Zhang ZY 2020 | −0.005 | 0.0512 | 19.1% | 1.00 [0.90, 1.10] |
| Zhao JP 2021 | 0.0477 | 0.0243 | 26.9% | 1.05 [1.00, 1.10] |
| **Subtotal (95% CI)** | | | **100.0%** | **1.09 [1.02, 1.17]** |
| Heterogeneity: Tau$^2$ = 0.00; Chi$^2$ = 30.67, df = 16 (P = 0.01); I$^2$ = 48% | | | | |
| Test for overall effect: Z = 2.41 (P = 0.02) | | | | |

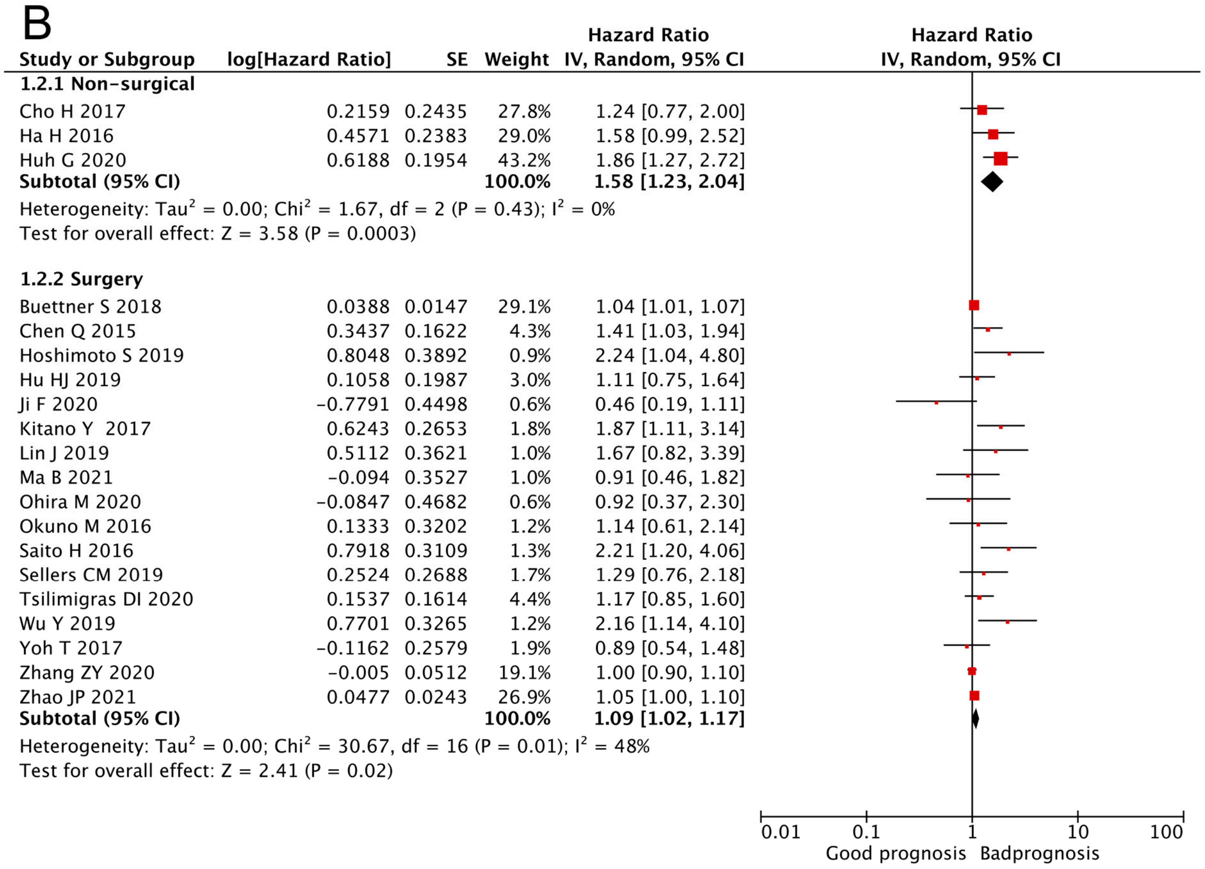


C

| Study or Subgroup | log[Hazard Ratio] | SE | Weight | Hazard Ratio IV, Random, 95% CI |
|---|---|---|---|---|
| **1.4.1 PLR>=150** | | | | |
| Buettner S 2018 | 0.0388 | 0.0147 | 28.7% | 1.04 [1.01, 1.07] |
| Hoshimoto S 2019 | 0.8048 | 0.3892 | 2.7% | 2.24 [1.04, 4.80] |
| Hu HJ 2019 | 0.1058 | 0.1987 | 8.3% | 1.11 [0.75, 1.64] |
| Ji F 2020 | −0.7791 | 0.4498 | 2.1% | 0.46 [0.19, 1.11] |
| Kitano Y 2017 | 0.6243 | 0.2653 | 5.3% | 1.87 [1.11, 3.14] |
| Okuno M 2016 | 0.1333 | 0.3202 | 3.9% | 1.14 [0.61, 2.14] |
| Saito H 2016 | 0.7918 | 0.3109 | 4.1% | 2.21 [1.20, 4.06] |
| Sellers CM 2019 | 0.2524 | 0.2688 | 5.2% | 1.29 [0.76, 2.18] |
| Tsilimigras DI 2020 | 0.1537 | 0.1614 | 11.0% | 1.17 [0.85, 1.60] |
| Wu Y 2019 | 0.7701 | 0.3265 | 3.7% | 2.16 [1.14, 4.10] |
| Zhang ZY 2020 | −0.005 | 0.0512 | 24.9% | 1.00 [0.90, 1.10] |
| **Subtotal (95% CI)** | | | **100.0%** | **1.17 [1.02, 1.33]** |
| Heterogeneity: Tau$^2$ = 0.02; Chi$^2$ = 24.95, df = 10 (P = 0.005); I$^2$ = 60% | | | | |
| Test for overall effect: Z = 2.27 (P = 0.02) | | | | |
| **1.4.2 PLR<150** | | | | |
| Chen Q 2015 | 0.3437 | 0.1622 | 15.4% | 1.41 [1.03, 1.94] |
| Cho H 2017 | 0.2159 | 0.2435 | 10.0% | 1.24 [0.77, 2.00] |
| Ha H 2016 | 0.4571 | 0.2383 | 10.3% | 1.58 [0.99, 2.52] |
| Huh G 2020 | 0.6188 | 0.1954 | 12.9% | 1.86 [1.27, 2.72] |
| Lin J 2019 | 0.5112 | 0.3621 | 5.7% | 1.67 [0.82, 3.39] |
| Ma B 2021 | −0.094 | 0.3527 | 5.9% | 0.91 [0.46, 1.82] |
| Ohira M 2020 | −0.0847 | 0.4682 | 3.7% | 0.92 [0.37, 2.30] |
| Yoh T 2017 | −0.1162 | 0.2579 | 9.3% | 0.89 [0.54, 1.48] |
| Zhao JP 2021 | 0.0477 | 0.0243 | 26.7% | 1.05 [1.00, 1.10] |
| **Subtotal (95% CI)** | | | **100.0%** | **1.25 [1.03, 1.51]** |
| Heterogeneity: Tau$^2$ = 0.03; Chi$^2$ = 16.89, df = 8 (P = 0.03); I$^2$ = 53% | | | | |
| Test for overall effect: Z = 2.31 (P = 0.02) | | | | |

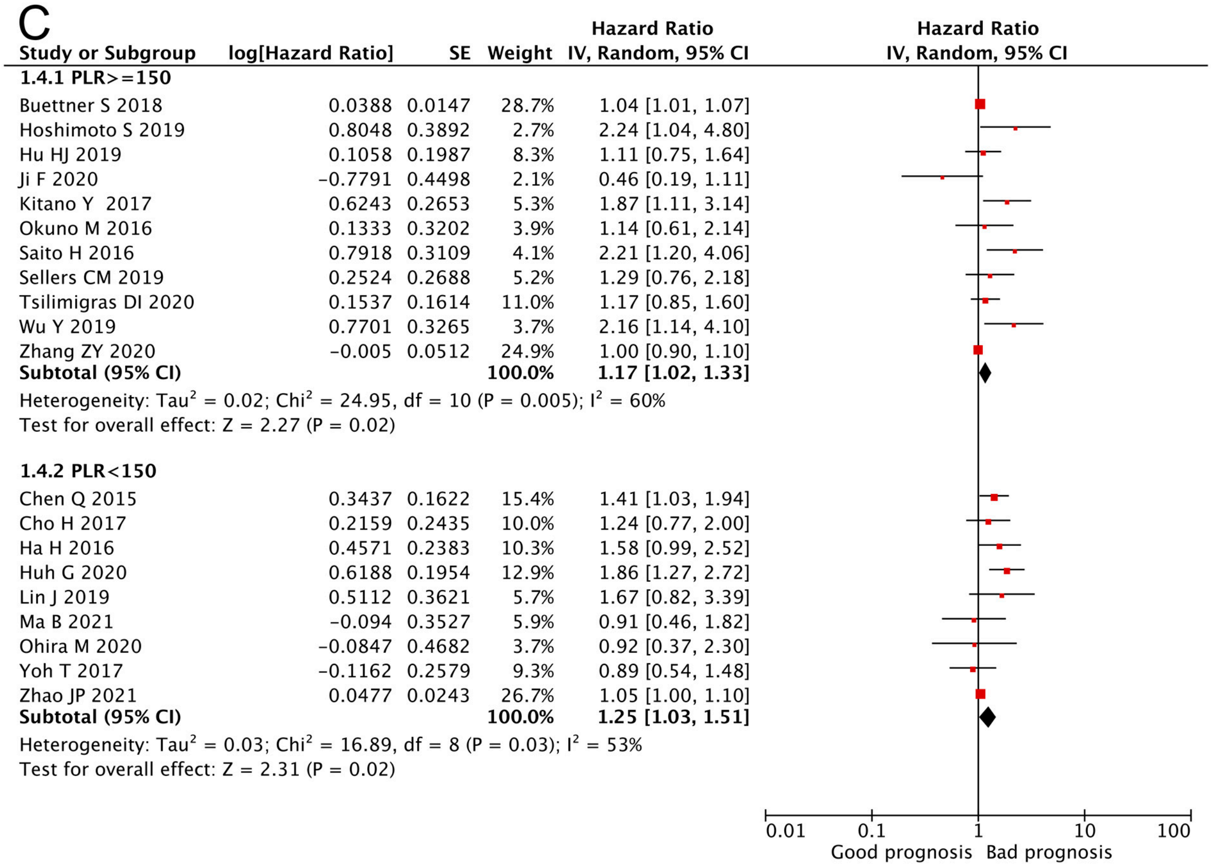

D

| Study or Subgroup | log[Hazard Ratio] | SE | Weight | Hazard Ratio IV, Random, 95% CI |
|---|---|---|---|---|
| **1.5.1 Sample >= 200** | | | | |
| Buettner S 2018 | 0.0388 | 0.0147 | 48.2% | 1.04 [1.01, 1.07] |
| Chen Q 2015 | 0.3437 | 0.1622 | 3.1% | 1.41 [1.03, 1.94] |
| Cho H 2017 | 0.2159 | 0.2435 | 1.4% | 1.24 [0.77, 2.00] |
| Ha H 2016 | 0.4571 | 0.2383 | 1.5% | 1.58 [0.99, 2.52] |
| Ji F 2020 | −0.7791 | 0.4498 | 0.4% | 0.46 [0.19, 1.11] |
| Lin J 2019 | 0.5112 | 0.3621 | 0.6% | 1.67 [0.82, 3.39] |
| Okuno M 2016 | 0.1333 | 0.3202 | 0.8% | 1.14 [0.61, 2.14] |
| Sellers CM 2019 | 0.2524 | 0.2688 | 1.2% | 1.29 [0.76, 2.18] |
| Tsilimigras DI 2020 | 0.1537 | 0.1614 | 3.1% | 1.17 [0.85, 1.60] |
| Zhao JP 2021 | 0.0477 | 0.0243 | 39.8% | 1.05 [1.00, 1.10] |
| **Subtotal (95% CI)** | | | **100.0%** | **1.07 [1.01, 1.13]** |
| Heterogeneity: $Tau^2 = 0.00$; $Chi^2 = 13.20$, df = 9 (P = 0.15); $I^2 = 32\%$ | | | | |
| Test for overall effect: Z = 2.28 (P = 0.02) | | | | |
| **1.5.2 Sample < 200** | | | | |
| Hoshimoto S 2019 | 0.8048 | 0.3892 | 6.8% | 2.24 [1.04, 4.80] |
| Huh G 2020 | 0.6188 | 0.1954 | 12.6% | 1.86 [1.27, 2.72] |
| Hu HJ 2019 | 0.1058 | 0.1987 | 12.5% | 1.11 [0.75, 1.64] |
| Kitano Y 2017 | 0.6243 | 0.2653 | 10.2% | 1.87 [1.11, 3.14] |
| Ma B 2021 | −0.094 | 0.3527 | 7.7% | 0.91 [0.46, 1.82] |
| Ohira M 2020 | −0.0847 | 0.4682 | 5.4% | 0.92 [0.37, 2.30] |
| Saito H 2016 | 0.7918 | 0.3109 | 8.8% | 2.21 [1.20, 4.06] |
| Wu Y 2019 | 0.7701 | 0.3265 | 8.4% | 2.16 [1.14, 4.10] |
| Yoh T 2017 | −0.1162 | 0.2579 | 10.4% | 0.89 [0.54, 1.48] |
| Zhang ZY 2020 | −0.005 | 0.0512 | 17.2% | 1.00 [0.90, 1.10] |
| **Subtotal (95% CI)** | | | **100.0%** | **1.38 [1.07, 1.77]** |
| Heterogeneity: $Tau^2 = 0.10$; $Chi^2 = 28.68$, df = 9 (P = 0.0007); $I^2 = 69\%$ | | | | |
| Test for overall effect: Z = 2.46 (P = 0.01) | | | | |

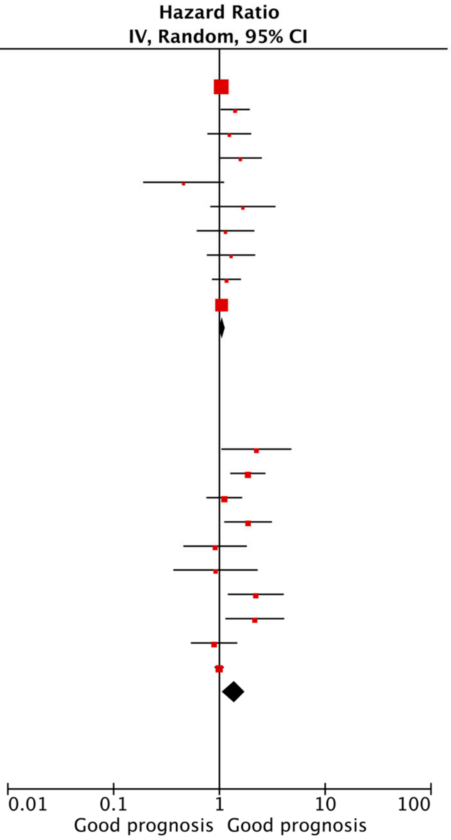


E

| Study or Subgroup | log[Hazard Ratio] | SE | Weight | Hazard Ratio IV, Random, 95% CI |
|---|---|---|---|---|
| **1.6.2 Age >= 60** | | | | |
| Hoshimoto S 2019 | 0.8048 | 0.3892 | 6.9% | 2.24 [1.04, 4.80] |
| Huh G 2020 | 0.6188 | 0.1954 | 16.8% | 1.86 [1.27, 2.72] |
| Hu HJ 2019 | 0.1058 | 0.1987 | 16.5% | 1.11 [0.75, 1.64] |
| Lin J 2019 | 0.5112 | 0.3621 | 7.7% | 1.67 [0.82, 3.39] |
| Okuno M 2016 | 0.1333 | 0.3202 | 9.3% | 1.14 [0.61, 2.14] |
| Saito H 2016 | 0.7918 | 0.3109 | 9.7% | 2.21 [1.20, 4.06] |
| Sellers CM 2019 | 0.2524 | 0.2688 | 11.7% | 1.29 [0.76, 2.18] |
| Wu Y 2019 | 0.7701 | 0.3265 | 9.0% | 2.16 [1.14, 4.10] |
| Yoh T 2017 | −0.1162 | 0.2579 | 12.4% | 0.89 [0.54, 1.48] |
| **Subtotal (95% CI)** | | | **100.0%** | **1.48 [1.18, 1.85]** |
| Heterogeneity: $Tau^2 = 0.04$; $Chi^2 = 12.42$, df = 8 (P = 0.13); $I^2 = 36\%$ | | | | |
| Test for overall effect: Z = 3.38 (P = 0.0007) | | | | |
| **1.6.3 Age <60** | | | | |
| Buettner S 2018 | 0.0388 | 0.0147 | 37.6% | 1.04 [1.01, 1.07] |
| Chen Q 2015 | 0.3437 | 0.1622 | 3.0% | 1.41 [1.03, 1.94] |
| Cho H 2017 | 0.2159 | 0.2435 | 1.4% | 1.24 [0.77, 2.00] |
| Ha H 2016 | 0.4571 | 0.2383 | 1.5% | 1.58 [0.99, 2.52] |
| Ji F 2020 | −0.7791 | 0.4498 | 0.4% | 0.46 [0.19, 1.11] |
| Kitano Y 2017 | 0.6243 | 0.2653 | 1.2% | 1.87 [1.11, 3.14] |
| Ma B 2021 | −0.094 | 0.3527 | 0.7% | 0.91 [0.46, 1.82] |
| Ohira M 2020 | −0.0847 | 0.4682 | 0.4% | 0.92 [0.37, 2.30] |
| Tsilimigras DI 2020 | 0.1537 | 0.1614 | 3.1% | 1.17 [0.85, 1.60] |
| Zhang ZY 2020 | −0.005 | 0.0512 | 18.4% | 1.00 [0.90, 1.10] |
| Zhao JP 2021 | 0.0477 | 0.0243 | 32.3% | 1.05 [1.00, 1.10] |
| **Subtotal (95% CI)** | | | **100.0%** | **1.06 [1.00, 1.12]** |
| Heterogeneity: $Tau^2 = 0.00$; $Chi^2 = 16.80$, df = 10 (P = 0.08); $I^2 = 40\%$ | | | | |
| Test for overall effect: Z = 1.95 (P = 0.05) | | | | |

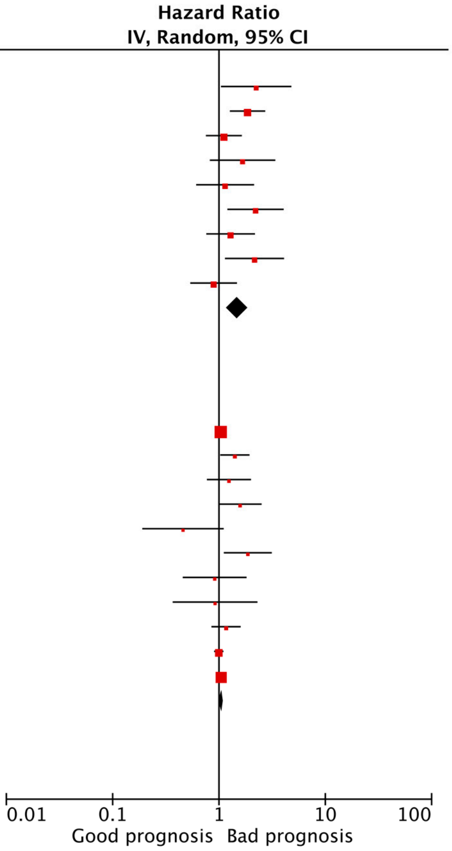

**Figure S1.** Stratified forest plots of the association between the PLR and OS in CCA patients. Subgroup analysis of the association between the PLR and OS based on cancer type (A), treatment (B), PLR cut-off values (C), sample size (D) and age (E). A random-effects model was used. OS, overall survival; PLR, platelet-to-lymphocyte ratio.

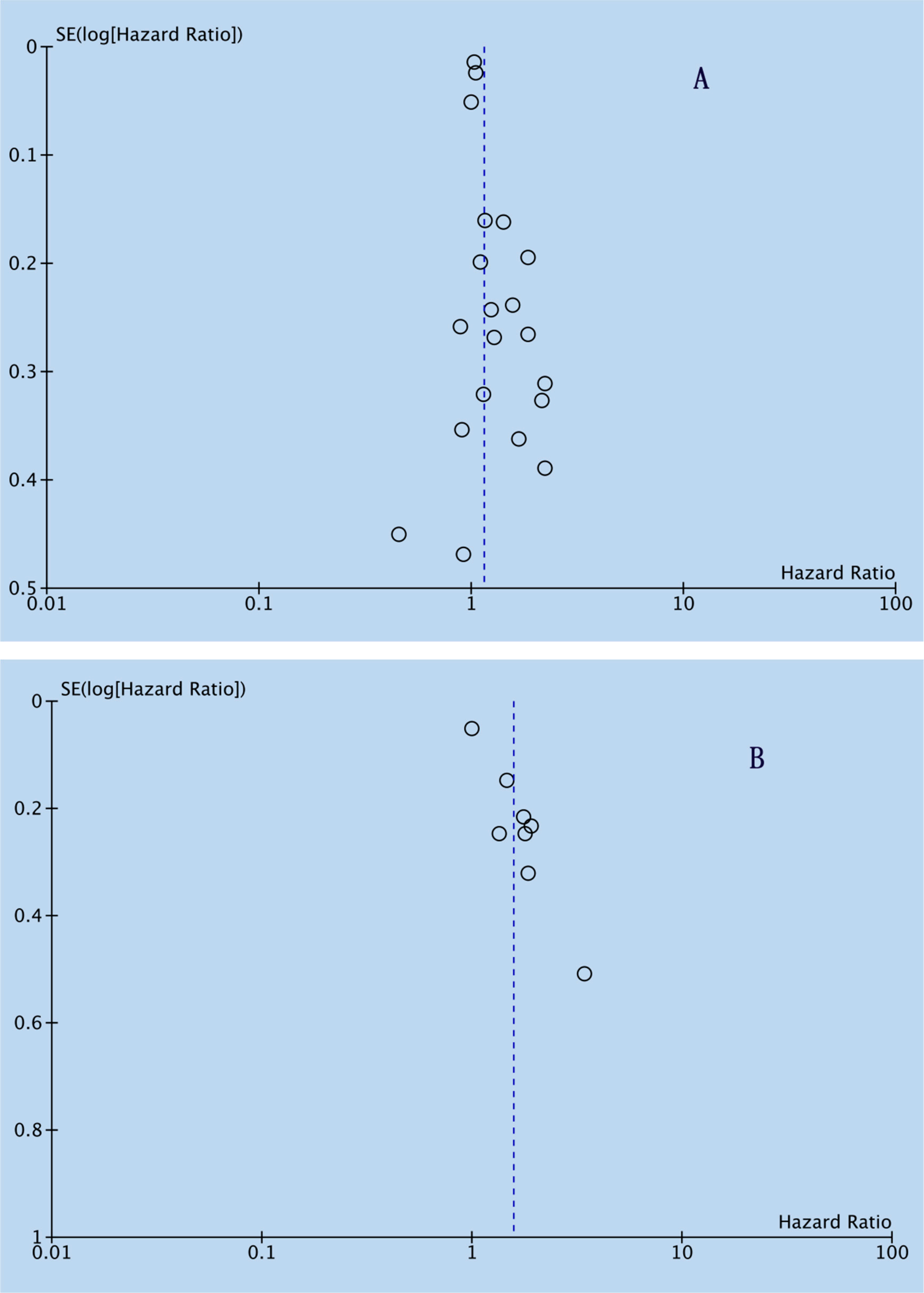


**Figure S2. Funnel plot of the meta-analysis of PLR in OS(A) and DFS(B).**
